# Immunolocalization of Influenza A Virus and Markers of Inflammation in the Human Parkinson's Disease Brain

**DOI:** 10.1371/journal.pone.0020495

**Published:** 2011-05-31

**Authors:** Troy T. Rohn, Lindsey W. Catlin

**Affiliations:** Department of Biology, Boise State University, Boise, Idaho, United States of America; University of Hong Kong, Hong Kong

## Abstract

Although much is known regarding the molecular mechanisms leading to neuronal cell loss in Parkinson's disease (PD), the initiating event has not been identified. Prevailing theories including a chemical insult or infectious agent have been postulated as possible triggers, leading to neuroinflammation. We present immunohistochemical data indicating the presence of influenza A virus within the substantia nigra pars compacta (SNpc) from postmortem PD brain sections. Influenza A virus labeling was identified within neuromelanin granules as well as on tissue macrophages in the SNpc. Further supporting a role for neuroinflammation in PD was the identification of T-lymphocytes that colocalized with an antibody to caspase-cleaved Beclin-1 within the SNpc. The presence of influenza A virus together with macrophages and T-lymphocytes may contribute to the neuroinflammation associated with this disease.

## Introduction

Parkinson's disease (PD) is progressive neurodegenerative disorder characterized by extrapyramidal movement disorders that manifest as rigidity, resting tremor and postural instability [1]. Neuronal cell loss occurs in the substantia nigra pars compacta (SNpc) and is associated with the presence of Lewy body inclusions that are comprised principally of aggregated alpha-synuclein [2]. Although the molecular steps leading to alpha-synuclein aggregation and neuronal cell loss are well delineated in PD, the initiating trigger to these events remains unknown. Two prevailing theories involving a chemical insult or infectious agent have emerged as the leading candidates for serving as external triggers for initiating the pathology underlying idiopathic PD. A viral etiology for PD is based largely on epidemiological studies indicating a possible coincidence of PD with influenza flu pandemics, most notably the 1918–1919 “Spanish” influenza outbreak [3]. In addition, reports of clusters in PD have also correlated viral agents as initiators of parkinsonism [4], [5].

Experimental evidence from animal models has also supported a possible role of viral agents as participating in the initiating events underlying PD. For example, intracerebral injection of a neurovirulent strain of influenza A virus into mice indicated a preferential localization of the virus within the substantia nigra [6]. More recently, a study by Jang et. al. demonstrated the infection of mice with the H5N1 avian influenza virus resulted in CNS infiltration, activation of microglia and alpha-synuclein phosphorylation and aggregation that persisted long after the resolution of the infection [7].

Despite these reports correlating a viral infection as a triggering mechanism in PD, little direct evidence exists for the presence of influenza A virus in PD brains. In the current study, we now report immunohistochemical evidence for influenza A viral particles in the human PD brain. The majority of labeling we observed for influenza A was found on macrophages located in the substantia nigra. In addition, we also provide evidence of other immune cell mediators, T-lymphocytes, present in the human PD brain. Our results suggest the localization of influenza A virus within the substantia nigra of PD cases, along with immune cells that together may contribute to the neuroinflammation associated with this disease.

## Results

**Figure 1:**
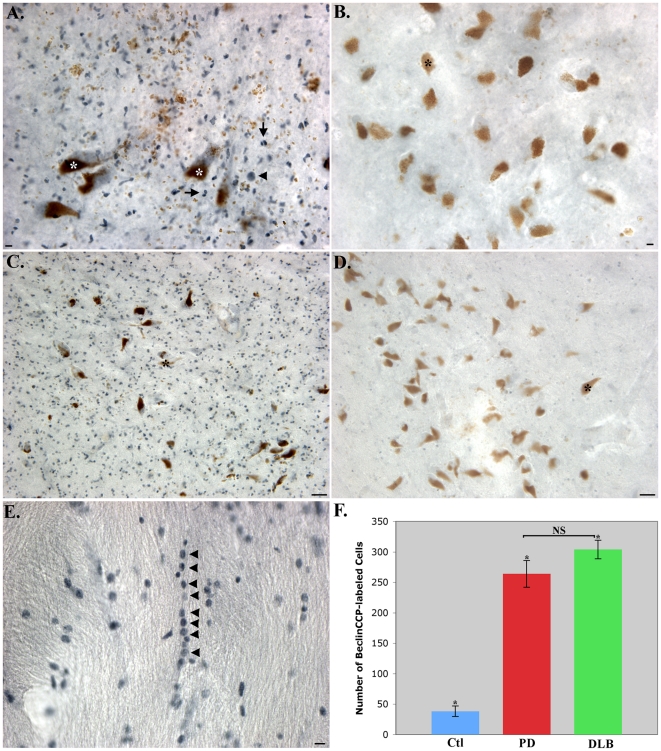
Caspase-cleaved Beclin-1 in Parkinson's and dementia with Lewy body disease. (**A–D**): High (A and B) and low magnification (C and D), of representative single-labeling (blue) from a Parkinson's cases utilizing the BeclinCCP antibody illustrating staining of numerous small cells (<10 µm) in the SNpc (arrows, Fig. 1A) along with the staining of a single Lewy body (arrowhead, Fig. 1A). Comparative staining in representative age-matched control cases showing a general lack of staining with the BeclinCCP antibody (B and D). Brown structures (A–D) represent neuromelanin (asterisks), typical of neurons in the SNpc. (**E**): Representative labeling in a DLB case illustrating the “pearls-on-a-string” labeling with BeclinCCP in white matter within the SN (E, arrowheads). (**F**): Quantification of the number of BeclinCCP-positive cells within the SNpc for age-matched control cases (blue bar), PD cases, (red bar) and DLB cases (green bar). Results indicated a significant increase in the number of BeclinCCP-positive cells in both PD and DLB over control cases. Data represent the average (±S.E.M.) of three different fields taken with a 40× objective from five different cases. NS = no significant difference between PD and DLB cases (p = 0.331). *PD indicates significant difference between PD and control cases (p = 0.0005), and *DLB indicates significant difference between DLB and control cases (p = 0.0001). Scale bars are 10 µm in A, B, and E and 50 µm in C and D.

**Figure 2:**
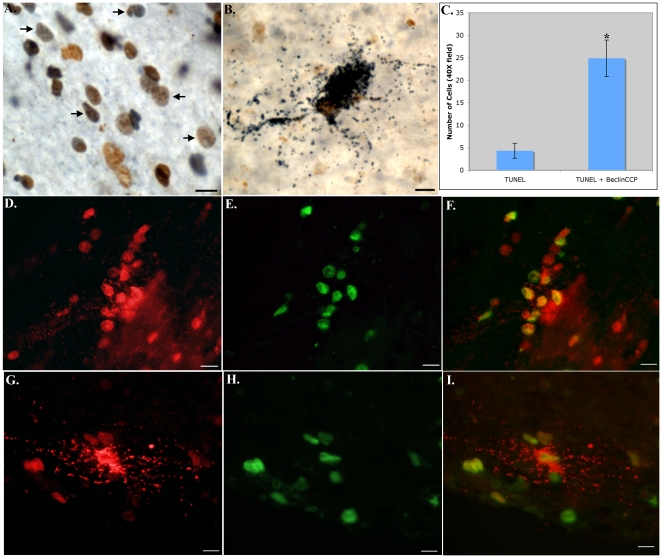
Co-localization of the BeclinCCP antibody with TUNEL labeling, a marker for apoptosis. (**A and B**): Representative bright-field double-labeling utilizing the BeclinCCP antibody (blue) and TUNEL (DAB, brown) revealed the co-localization within small cells (arrows, A) and within a degenerating astrocyte (B) in SNpc of representative Parkinson's cases. (**D–I**): Representative immunofluorescence double-labeling within the SN utilizing BeclinCCP (red) and TUNEL (green) in a representative PD case (D–F) or DLB (G–I). Panel G illustrates the staining of a degenerating astrocyte that co-localized with TUNEL (Panel I). (**C**): Panel C depicts quantitative immunofluorescence analysis indicating that approximately 85% of TUNEL-positive cells co-localized with the BeclinCCP antibody (p = 1.38×10^−5^). Scale bars are 10 µm.

Case demographics for PD cases are presented in **Table S1** and age at death was not significantly different between PD (mean, 74.3±11.3), DLB (mean, 76.7±4.04) and controls (mean, 73.9±6.81). Representative pathology in PD cases including the presence of Lewy bodies and neurites, gliosis, and loss of dopaminergic neurons is depicted in **Figure S1**. The initial goal of our study was to examine whether the autophagic protein, Beclin-1, is caspase-cleaved in the PD brain. The Beclin-1 protein is essential for the proper execution of autophagy, a process that regulates the turnover of cellular constituents and evidence supports this vital function may be disrupted in PD [8], [9]. Previous studies have supported a loss of function of Beclin-1 due to proteolytic cleavage by caspases [10]. PD cases were analyzed for the presence of caspase-cleaved Beclin-1 following application of a site-directed caspase-cleavage antibody. We had previously used this antibody (herein termed the Beclin caspase-cleavage product (CCP) antibody) to demonstrate the caspase-cleavage of Beclin-1 within degenerating astrocytes and tangles of the Alzheimer's disease (AD) brain [11]. The BeclinCCP antibody is specific for the caspase-cleaved fragment of Beclin-1 *in situ* [11]. Surprisingly, the application of the BeclinCCP antibody in either PD or dementia with Lewy body (DLB) cases revealed labeling of numerous, small cells (<10 µm), throughout the SN (Fig. 1). Labeling was also observed in Lewy bodies (Fig. 1A), degenerating astrocytes (Fig. 2B), and apparent oligodendrocytes (Fig. 1E). There was a significant increase in the number of BeclinCCP-labeled cells as compared to age-matched controls (Fig. 1F).

Because the BeclinCCP antibody is a marker for caspase activation [11], we next determined the extent of co-localization of this antibody with TUNEL, a marker for apoptosis. In this regard, co-localization experiments demonstrated that 85% of TUNEL-positive cells co-localized with the BeclinCCP antibody (p = 1.38×10^−5^, ±S.E.M.), supporting the idea that these cells were undergoing apoptosis (Fig. 2).

**Figure 3:**
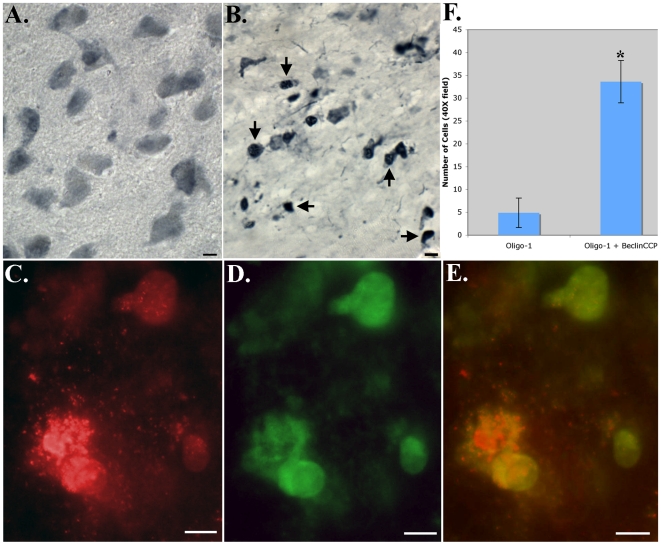
Degenerating oligodendrocytes co-localize with caspase-cleaved Beclin-1 in PD. (**A and B**): Representative labeling in control or PD cases utilizing mAB anti-Olig1 indicated staining of well-defined, healthy oligodendrocytes in age-matched control cases (A), as compared to PD cases where labeling was identified on oligodendrocytes exhibiting shrunken cell bodies (B). (**C–E**): Immunofluorescence double labeling in a representative PD case indicated the co-localization of the BeclinCCP antibody (red, C) with anti-Olig1 (green, D). Panel E displays the overlap image for both antibodies indicating the labeling of the BeclinCCP antibody within degenerating oligodendrocytes (yellow, E). **F**): Panel F depicts quantitative analysis indicating that approximately 87% of anti-Olig1-positive cells co-localized with the BeclinCCP antibody (p = 1.46×10^−6^). All scale bars are 10 µm.

Due to the pattern of labeling of the BeclinCCP antibody in SN white matter (Fig. 1E), it was predicted that labeled cells were oligodendrocytes. We observed staining of cells of similar size and morphology as compared to BeclinCCP staining following application of an oligodendrocyte antibody, anti-Olig1 in PD cases. Thus, single-labeling experiments with anti-Oligo1 indicated labeling of oligodendrocytes exhibiting shrunken cells bodies in PD cases, indicative of cells undergoing apoptosis (Fig. 3B), whereas in age-matched control cases, labeling of well-defined oligodendrocytes was observed (Fig. 3A). That the BeclinCCP antibody was labeling oligodendrocytes was confirmed following co-localization immunofluorescence experiments utilizing anti-Olig1 and BeclinCCP (Fig. 3C–E). Under these experimental conditions, quantitative analysis demonstrated that 87% of anti-Oligo1-positive cells co-localized with the BeclinCCP antibody (p = 1.46×10^−6^, ±S.E.M.) (Fig. 3F).

**Figure 4:**
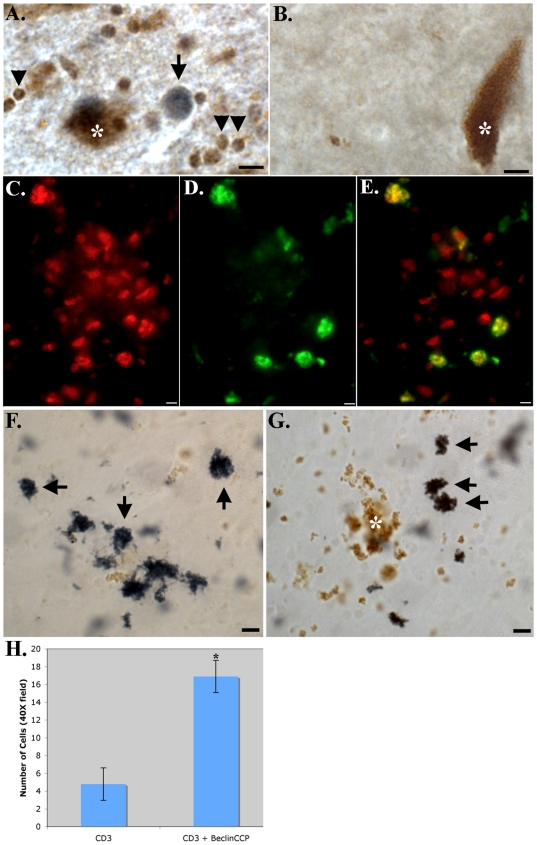
Caspase-cleaved Beclin-1 within T-lymphocytes in the PD brain. (**A and B**): Bright-field double labeling in the SNpc of a representative PD case (A) or control (B) illustrating co-localization of the BeclinCCP antibody (blue) with mAB CD3, a marker for T-lymphocytes (brown). Note the size and co-localization of both markers in cells (arrowheads, A), and in addition the single labeling of BeclinCCP within an apparent Lewy body (arrow, A). There was a general lack of labeling of both markers in control cases (B). Brown structures (A, B, F, and G) represent neuromelanin (asterisks), typical of neurons in the SNpc. (**C–E**): Representative immunofluorescence double labeling employing the BeclinCCP antibody (red, C) and mAB CD3 (green, D) in the SNpc of a representative PD case, with the overlapped image for both markers (yellow, E). (**F and G**): Representative staining in the SNpc of a PD case with anti-CD4 in (F) and anti-CD8 (G), markers for helper T-lymphocytes and cytotoxic T-lymphocytes, respectively (arrows). (**H**): Panel H depicts quantitative analysis indicating that approximately 78% of CD3-positive cells co-localized with the BeclinCCP antibody (p = 7.08×10^−5^). Scale bars represent 10 µm.

Single labeling experiments with the anti-oligodendrocyte antibody revealed little labeling of oligodendrocytes within the SNpc (**Figure S2**). Therefore, we hypothesized that an additional cell type was being labeled with the BeclinCCP antibody in this region. Based on the size and morphology of the cells labeled, we performed co-localization experiments with BeclinCCP and anti-CD3, a marker for T-lymphocytes. Co-localization of these two antibodies using bright field microscopy was evident within the SNpc in all PD cases examined (Fig. 4A), which was largely absent in age-matched control cases (Fig. 4B). Due to the lack of color separation using bright-field microscopy, additional co-localization experiments were undertaken using immunofluorescence (Fig. 4C–E). For immunofluorescence experiments, of those cells that were CD3-positive, 78% of these cells also were labeled with the BeclinCCP antibody (p = 7.08×10^−5^, ±S.E.M.) (Fig. 4H). The presence of caspase-cleaved Beclin-1 within T-lymphocytes would suggest caspase activation, a general feature of T-lymphocytes undergoing activation [12]. The presence of CD3-positive cells itself is not indicative of neuroinflammation, therefore, further experiments were performed to assess the type of T-lymphocytes using CD4 and CD8 antibodies. We detected the presence of both CD4+ and CD8+ T-lymphocytes in PD cases, and the staining profile was similar for both antibodies (Fig. 4F and G). In addition, staining of cells was often in areas of depigmentation (Fig. 4G), which may be indicative of neuroinflammation [13]. These results suggest the presence of both helper and cytotoxic T-lymphocytes in the SNpc of the PD brain, supporting previous studies [14], [15], [16].

**Figure 5:**
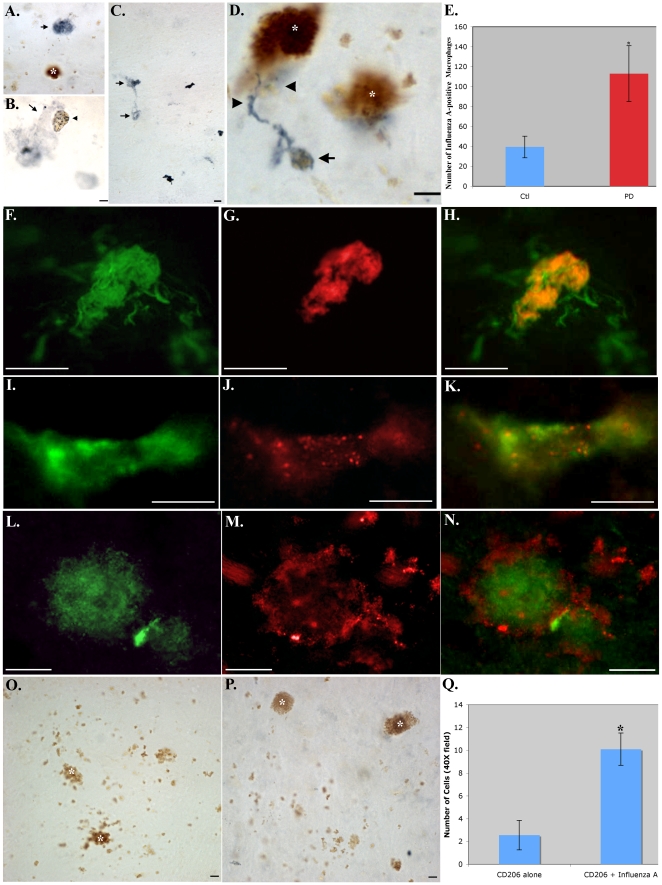
Evidence for influenza A virus on macrophages within the PD brain. (**A–C**): Representative staining (blue) within the SNpc of the PD brain utilizing mAB anti-influenza A virus antibody (35–481) depicting staining within large globular cells consistent with a morphology of macrophages (arrows, A–C). Panel B depicts staining within a macrophage-like cell extending an apparent pseudopod engulfing a smaller circular structure containing neuromelanin with punctate blue labeling (arrowhead, B). (**D**): Representative anti-influenza A virus staining (blue) in a control case identifying a single macrophage-like cell (arrowheads) surrounding a particle of neuromelanin material (arrow). (**E**): Quantitative analysis of the number of influenza A-positive macrophages labeled in Ctl (n = 5) or PD cases (n = 4) indicated a significant increase in PD cases (±S.D., p-value = 0.01). (**F–K**): Double labeling immunofluorescence in the SNpc of representative PD cases utilizing mAB anti-CD206, a macrophage marker (green, F and I), along with anti-influenza A virus labeling (35–481) (red, G and J) with the overlap image (yellow/orange, H and K). Two types of labeling were observed: In rounder macrophages (F), influenza A staining was more localized and homogenous (G). In perivascular regions, macrophages were more elongated (I) and influenza A labeling was clearly punctated in appearance (J). (**L–N**): Identical to Panels F–K, except with the use of a different antibody marker to both macrophages (CD14, green, L) and influenza A virus (35–483, red, M), with overlap image shown in Panel N. Punctate labeling of influenza A virus was observed on the surface of macrophages. (**O**): Control experiment showing lack of influenza A blue labeling in the absence of primary antibody. (**P**): Representative staining in a PD case utilizing an antibody against influenza B, indicating a general lack of blue labeling with this antibody. (**Q**): Panel Q depicts quantitative analysis indicating that approximately 80% of anti-CD206-positive cells co-localized with the anti-influenza A virus antibody (p = 4.55×10^−6^). Brown structures (A, D, O, and P) represent neuromelanin (asterisks), typical of neurons in the SNpc. All scale bars are 10 µm.

Because T-lymphocytes are known to respond to viral antigens and due to the long-standing idea that the triggering mechanism in PD involves an infectious agent [6], we examined PD cases utilizing an influenza A virus antibody known to detect numerous strains of the virus (Prosci, catalogue #35-481). We observed labeling on apparent macrophages in both PD (Fig. 5A–C) and control cases (Fig. 5D), although there was a significant increase (p = 0.01) in labeled macrophages of PD cases (Fig. 5E). Evidence for influenza A labeling was observed in all five PD cases examined and staining was absent in the presence of secondary antibody only (Fig. 5O). In addition, we examined PD cases for the presence of influenza B viral proteins, a virus not linked to PD and more commonly found in children than the elderly [17]. We were unable to detect any influenza B labeling in PD cases (Fig. 5P), suggesting a lack of this infectious agent in the PD brain.

Double label immunofluorescence experiments confirmed the co-localization of influenza A on macrophages (CD206 antibody) (Fig. 5F–K). We also observed punctate labeling of the influenza A virus antibody within neuromelanin structures (Fig. 5B). Co-localization experiments demonstrated that 80% of Macrophage-positive cells co-localized with the influenza A virus antibody (p = 4.55×10^−6^, ±S.E.M.) (Fig. 5Q). These results were confirmed using additional, distinct antibodies to macrophages (CD14) and influenza A virus (ProSci #35-483) (Fig. 5L–N). Similar results were also observed in DLB cases (**Figure S3**).

**Figure 6:**
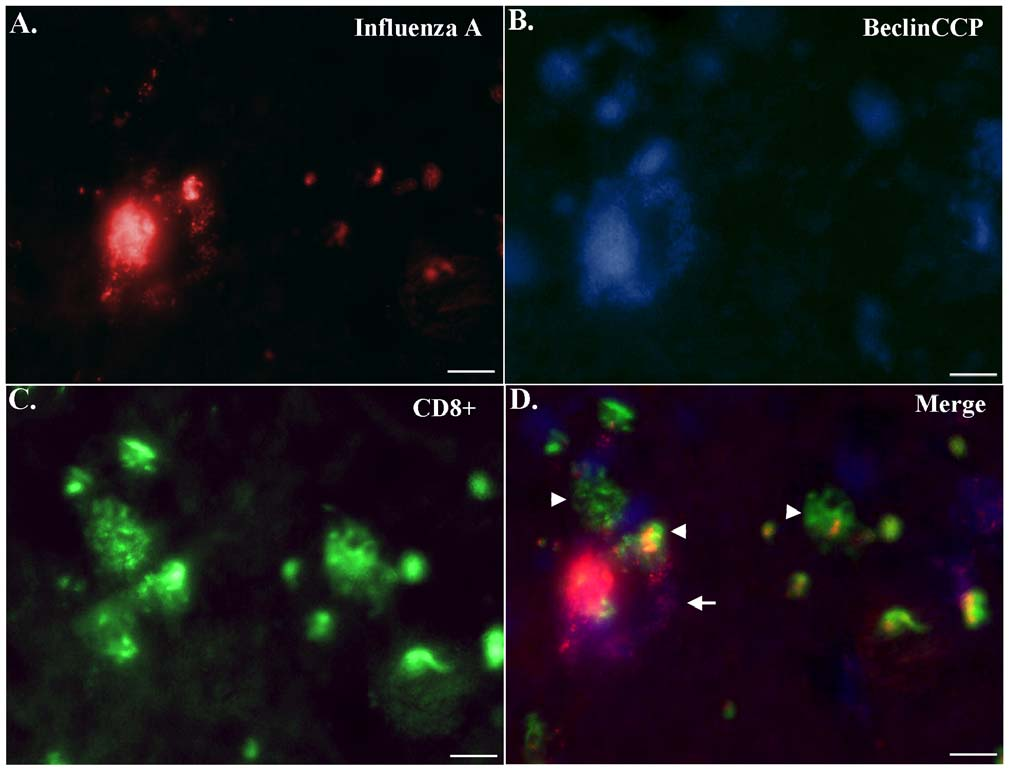
Influenza A-positive macrophages co-localize with caspase-cleaved Beclin-1 and are surrounded by cytotoxic T-lymphocytes. Representative immunofluorescence triple labeling in a PD case was undertaken using three different antibodies: (**A**) anti-influenza A (red), (**B**) BeclinCCP (blue), and (**C**) anti-CD8 (green). (**D**): Panel D displays the overlapped image for all three antibodies indicating the presence of influenza A viral proteins on an apparent macrophage displaying diffuse BeclinCCP labeling (arrow, D). In addition, numerous CD8+ cells are in close proximity to the macrophage and some of these cells demonstrated colocalization with the influenza A virus antibody (arrowheads, D). All scale bars are 10 µm.

In a final set of experiments, triple-labeling experiments were undertaken in representative PD cases to determine the localization of influenza A virus, BeclinCCP, and CD8+ T-lymphocytes (Fig. 6). The overlap image for the three antibodies indicated the presence of an apparent macrophage labeled with both anti-influenza A and BeclinCCP surrounded by CD8+ T-lymphocytes (Fig. 6D). In addition, co-localization of the influenza A virus within CD8+ T-lymphocytes was evident, and many of these cells tended to cluster around the macrophage (Fig. 6D). These results are suggestive of a possible interaction between cytotoxic T-lymphocytes and macrophages.

## Discussion

Several important findings can be drawn from the current immunohistochemical study in PD. The first important finding of the current study was the identification of apoptotic oligodendrocytes labeled by the Beclin-1 caspase-cleavage product antibody (BeclinCCP) in the white matter of the SN of PD and DLB cases. Many of the oligodendrocytes labeled with the BeclinCCP antibody displayed hallmark features of apoptosis including fragmentation of processes and shrunken cell bodies. Because the critical role oligodendrocytes play in myelination of axons in the CNS, the degeneration of oligodendrocytes may contribute to the extrapyramidal symptoms associated with PD.

A second finding was the presence of T-lymphocytes in the PD brain. The infiltration of T-lymphocytes, in an attempt to mitigate a viral infection, may help spare neuronal loss in PD. On the other hand, emerging data implicates inflammatory processes in the contribution of the pathophysiology underlying PD [18]. In this regard, several studies have identified the presence of T-lymphocytes within the SNpc in either the human PD brain or in animal models [14], [15], [16]. Our data indicated the presence of both helper and cytotoxic T-lymphocytes in PD cases, suggesting a possible CNS infiltration of peripheral immune cells. Therefore, our data supports a growing body of research suggesting that adaptive immune systems may play a critical role in the neurodegeneration associated with PD disease (for recent reviews see [19], [20]). In support of this is a recent study by Brochard et al. who reported that CD4+ T-lymphocytes are cytotoxic in a mouse model of PD and that invading CD4+ T-lymphocytes contributes to dopamine neurodegeneration through Fas/FasL pathway [15].

A third finding of the current study was the presence of influenza A viral proteins within the SNpc, which supports the epidemiological data that has linked this virus to PD [3], [4], [5]. We found the presence of influenza A labeling in all seven PD and five DLB cases examined. An important caveat of the current student is the relatively small sample size for data analysis. It is possible that we coincidently chose five DLB and seven PD cases that were positive for influenza A viral proteins. It should be noted that immunohistochemical reports have been published that were negative for presence of influenza A viral particles or nucleic acids in the PD brain [21], [22]. However, these studies, like ours, also consisted of a relatively small sample size of PD cases. Thus, future studies should be directed at testing for the presence of influenza A viral proteins utilizing a larger cohort to definitively document the presence or absence of this virus in the PD brain. Another important caveat of the present study is our findings do not prove influenza A virus causes PD, but is only present in the PD brain. It is possible that the cases we examined had seasonal influenza A virus infection shortly before they died, and the virus had no bearing on the course of the disease. The available clinical information on these patients was limiting, however, it is noteworthy that out of 7 PD cases, only one case could have possibly had an influenza A viral infection as the cause of death was pneumonia (**Table S1**).

Of particular interest was the finding that the majority of influenza A viral labeling was found on macrophages. Macrophages play a vital role in the immune system by phagocytosing, processing and presenting foreign antigens on their cell surface [23]. Macrophages are also known to stimulate T-lymphocytes and other immune cells to respond to pathogens [23]. We also identified punctate influenza A viral protein labeling within neuromelanin structures. In some cases, macrophages could be seen extending pseudopods around these structures, suggesting a role of macrophages in the clearance of this material. Previous studies have supported the hypothesis that influenza A viruses are neurotropic, i.e., they can travel into the nervous system following systemic infection where they preferentially localize to the substantia nigra [6]. It is interesting to speculate on whether influenza A viruses somehow hone in and take resident in neurons containing neuromelanin. This in turn could lead to the attraction, interaction, and finally activation of macrophages and T-lymphocytes. In support of this idea were results following triple-labeling experiments that indicated a possible interaction between influenza A virus-macrophages and cytotoxic T-lymphocytes (Fig. 6). Because macrophages are known to function as antigen-presenting cells that can stimulate cytotoxic T-lymphocytes, these results suggest a possible mechanism for the neuroinflammation associated with PD [24].

Although neuroinflammation is one outcome following activation of the immune system in the CNS, an equally important alternative are immune cells such as macrophages and T-lymphocytes are serving a neuroprotective role [25]. This apparent paradox underscores the delicate balance between either pathogenic or repair processes, which can be triggered by the immune response. For example, there is evidence indicating that the immune response is beneficial rather than damaging after brain damage. Thus, studies from Schwartz and colleagues suggest that T-lymphocytes can elicit CNS maintenance by releasing neuroprotective molecules such as BDNF in areas of brain injury or provide helpful signals to resident cells, such as microglia and astrocytes, which protect and promote the recovery of the brain [26]. In addition, macrophages may clear debris after myelin damage and when this is impeded, delayed regeneration occurs [27]. Finally, results utilizing an animal model of chronic neurodegeneration demonstrated that the presence of CD4+ T-lymphocytes provides supportive neuroprotection by modulating the trophic/cytotoxic balance of glia within the CNS [28]. Collectively, these studies emphasize that autoimmune responses in the CNS are not always destructive but, instead, are crucial for repair and regeneration.

Even taking into consideration that the immune system can serve a vital protective role in a number of acute and chronic neurodegenerative diseases, the overwhelming body of evidence suggest a role of neuroinflammation as a critical step underlying the pathological disease mechanisms associated with PD [24]. Because of this, and based on our present findings, one possible preventative mechanism to attenuate the progression of PD would be annual vaccinations against the influenza A virus. It is noteworthy, that the centers for disease control (CDC) currently recommends annual vaccinations against influenza A in all individuals over the age of 65 [29]. Although our results show the presence of influenza A viral proteins in the SN of the PD brain, our data do not answer directly the question as to whether the observed PD pathology is dependent on influenza A viral infection. It is possible that our small study cohort was infected shortly before death and that the viral infection on a single occasion was independent to the neuroinflammation and pathology associated with PD. It is not possible to draw such conclusions from immunohistochemical analysis using postmortem brain sections from affected individuals. However, animal models of PD do support a causal relationship between viral infection and pathology in PD. For example, a recent study by Jang et al. demonstrated the systemic infection of mice with the H5N1 influenza virus led to CNS penetration, microglia activation, alpha-synuclein phosphorylation and aggregation, and loss of dopaminergic neurons in the SNpc [7]. That the adaptive immune system can contribute directly to neurodegeneration in PD was shown in a mouse model of PD where the authors demonstrated that dopaminergic cell death was markedly attenuated in the absence of mature T-lymphocytes [15]. Taken together, these data support a model by which, perhaps due to a low-grade chronic viral infection in the area of the SN leads to the infiltration of immune cells including T-lymphocytes and macrophages. These immune cells may have a primary purpose of eradicating the infection, but in the process also lead to the activation of resident microglia and astrocytes which in turn damage neurons by “friendly” fire. The potential arsenal released by T-lymphocytes and macrophages that may contribute to neuroinflammation include cytokine IL-17, granzyme B, TNFα and various free radicals, all which may contribute to dopaminergic neuronal death [20]. Collectively, our data support the role of neuroinflammation as an underlying feature of PD and suggest that both annual vaccinations against influenza A virus and anti-inflammatory medications may be one strategy in the treatment of this disease.

Although we observed a significant increase in the number of influenza A-positive macrophages in PD cases, labeling was also observed in age-matched control cases. It may be that PD subjects are somehow more susceptible to the presence of influenza A virus infection compared to normals. This may be related to genetic susceptibility in PD that may confer selective vulnerability to influenza A virus infiltration into the SNpc. Further studies are required, however, to determine if influenza A directly contributes to the neuroinflammation and pathology underlying PD, or is simply coincidental to the disease process.

## Materials and Methods

### Antibody dilutions

The rabbit BeclinCCP (in house, 1∶100). The mouse anti-human CD14, macrophage marker (1∶50), mouse anti-human CD206, macrophage marker (1∶50), mouse anti-human CD3, T-lymphocyte marker (1∶50), mouse anti-human CD4, helper T-lymphocyte marker (1∶50), and mouse anti-human CD8 (1∶50), cytotoxic T-lymphocyte marker were all purchased from BD Pharmingen. The mouse anti-influenza A virus antibodies, catalogue numbers 35–481, or 35–483 (1∶50), were both purchased from ProSci Incorporated. The anti-influenza A virus antibodies are known to recognize the nucleoprotein of numerous strains. Of the two antibodies, 35–483 gave the most robust degree of staining. The anti-influenza B virus antibody (1∶50) was also from ProSci Incorporated. To assess apoptosis, the Apoptag peroxidase kit was employed according the manufacturer's instructions (Millipore). Anti-Tyrosine Hydroxylase, clone LNC1 (1∶100) was purchased from Millipore.

### Immunohistochemistry

Autopsy brain tissue from seven neuropathologically confirmed PD cases, five cases from neuropathologically confirmed dementia with Lewy bodies (DLB), and five neuropathologically normal cases were studied. Human brain tissue sections used in this study were provided by the Institute for Memory Impairments and Neurological Disorders at the University of California, Irvine. Free-floating 40 µm-thick sections were used for immunohistochemical studies as previously described [30]. No approval from Boise State University Institutional Review Board was obtained due to the exemption granted that all tissue sections were fixed and received from University of California, Irvine. Sections from the substantia nigra were selected for immunohistochemical analysis.

For single labeling, all sections were washed with 0.1 M Tris-buffered saline (TBS), pH 7.4, and then pretreated with 3% hydrogen peroxide in 10% methanol to block endogenous peroxidase activity. Sections were subsequently washed in TBS with 0.1% Triton X-100 (TBS-A) and then blocked for thirty minutes in TBS-A with 3% bovine serum albumin (TBS-B). Sections were further incubated overnight at room temperature in various primary antibodies as listed above. Following two washes with TBS-A and a wash in TBS-B, sections were incubated in anti-rabbit or mouse biotinylated anti-IgG (1 hour) and then in avidin biotin complex (1 hour) (ABC, Elite Immunoperoxidase, Vector Laboratories, Burlingame, CA, USA). Antibodies were visualized using Blue SG substrate (Vector Laboratories). No pretreatment antigen retrieval protocol was employed for any of the primary antibodies used in this study. For bright-field immunohistochemical double labeling, primary antibody labeling was detected using the brown DAB substrate (Vector Labs), while the second label was visualized using the Blue SG substrate (Vector Labs).

### Immunofluorescence Microscopy

Immunofluorescence studies were performed by incubating sections with primary antibody overnight at a room temperature, followed by secondary anti-rabbit or mouse biotinylated anti-IgG (1 hour) and then in ABC (1 hour). Visualization was accomplished by using a tyramide signal amplification kit (Molecular Probes, Eugene, OR) consisting of Alexa Fluor 488-labeled tyramide (green, Ex/Em = 495/519). For immunofluorescence co-localization studies, antigen visualization was accomplished using an Alexa fluor 488-labeled tyramide (green, Ex/Em = 495/519) for one label and streptavidin Alexa fluor 555 (red, Ex/Em = 555/565) for the second label, both from Invitrogen (Carlsbad, CA).

### Statistical analysis

To determine the percent co-localization, a semi-quantitative analysis was performed as described previously [11], [31], [32] by taking 40× immunofluorescence, overlapping images from three different fields in the SN in three separate PD cases. Capturing was accomplished by using a 2.5× photo eyepiece, a Sony high resolution CCD video camera (XC-77). As an example, to determine the percent co-localization between CD3 and BeclinCCP, photographs were analyzed by counting the number of CD3-labeled T-lymphocytes alone per 40× field for each case, and the number of cells labeled with both CD3 and BeclinCCP. Data are representative of the average number (±S.E.M.) of CD3 or CD3 co-localized with BeclinCCP in each 40× field (3 fields total for 3 different cases).

Statistical differences in this study were determined using Student's two-tailed T-test employing Microsoft Office Excel.

## Supporting Information

Figure S1 **Representative pathology in PD cases.** Panels A, C, and E depict images from representative PD cases, while Panels B, D, and F are from representative control cases. (**A and B**): The presence of Lewy bodies (arrows, A) and Lewy neurites in PD (arrowheads, A) that was absent in age-matched control sections (B). (**C and D**): Neuroinflammation in PD cases was revealed following the demonstration of massive gliosis that was absent in age-matched control cases (D). (**E and F**): Loss of dopaminergic neurons in PD cases was revealed following labeling with an anti-tyrosine hydroxylase antibody (E) compared to age-matched controls (F). Note also the general loss of pigmentation in PD cases (A and E). All antibody staining is shown in blue, while brown labeling depicts the presence of neuromelanin that is typical of neurons found in the SNpc. All scale bars represent 10 µm.
(DOC)

Figure S2 **Relative lack of oligodendrocyte labeling in the SNpc of PD.** Representation from a PD case in blue showing the relative lack of labeling using anti-Olig1 in the SNpc (B) as compared to the widespread staining observed utilizing the BeclinCCP antibody (A). Arrows in Panel B designate the few oligodendrocytes labeled by anti-Olig1 in the SNpc. Brown structures shown in Panels A and B represent neuromelanin, typical of neurons in the SNpc. Scale bars represent 10 µm.
(DOC)

Figure S3 **Evidence for influenza A virus on macrophages within the DLB brain.** Double-labeling immunofluorescence in the SNpc of a representative DLB case utilizing mAB anti-CD206, a macrophage marker (green, A) along with anti-influenza A virus labeling (35–481) (red, B) with the overlap image (yellow/orange, C). Scale bar represents 10 µm.
(DOC)

Table S1 (DOC)
