# Supplementary material for: Immunolocalization of Influenza A Virus and Markers of Inflammation in the Human Parkinson's Disease Brain
Source: PLoS One. 2011 May 31;6(5):e20495. doi: 10.1371/journal.pone.0020495 (PMC3105060; doi:10.1371/journal.pone.0020495)
Supplement: Figure S1 — Representative pathology in PD cases. Panels A, C, and E depict images from representative PD cases, while Panels B, D, and F are from representative control cases. (A and B): The presence of Lewy bodies (arrows, A) and Lewy neurites in PD (arrowheads, A) that was absent in age-matched control sections (B). (C and D): Neuroinflammation in PD cases was revealed following the demonstration of massive gliosis that was absent in age-matched control cases (D). (E and F): Loss of dopaminergic neurons in PD cases was revealed following labeling with an anti-tyrosine hydroxylase antibody (E) compared to age-matched controls (F). Note also the general loss of pigmentation in PD cases (A and E). All antibody staining is shown in blue, while brown labeling depicts the presence of neuromelanin that is typical of neurons found in the SNpc. All scale bars represent 10 µm. (DOC) [file pone.0020495.s001.doc]

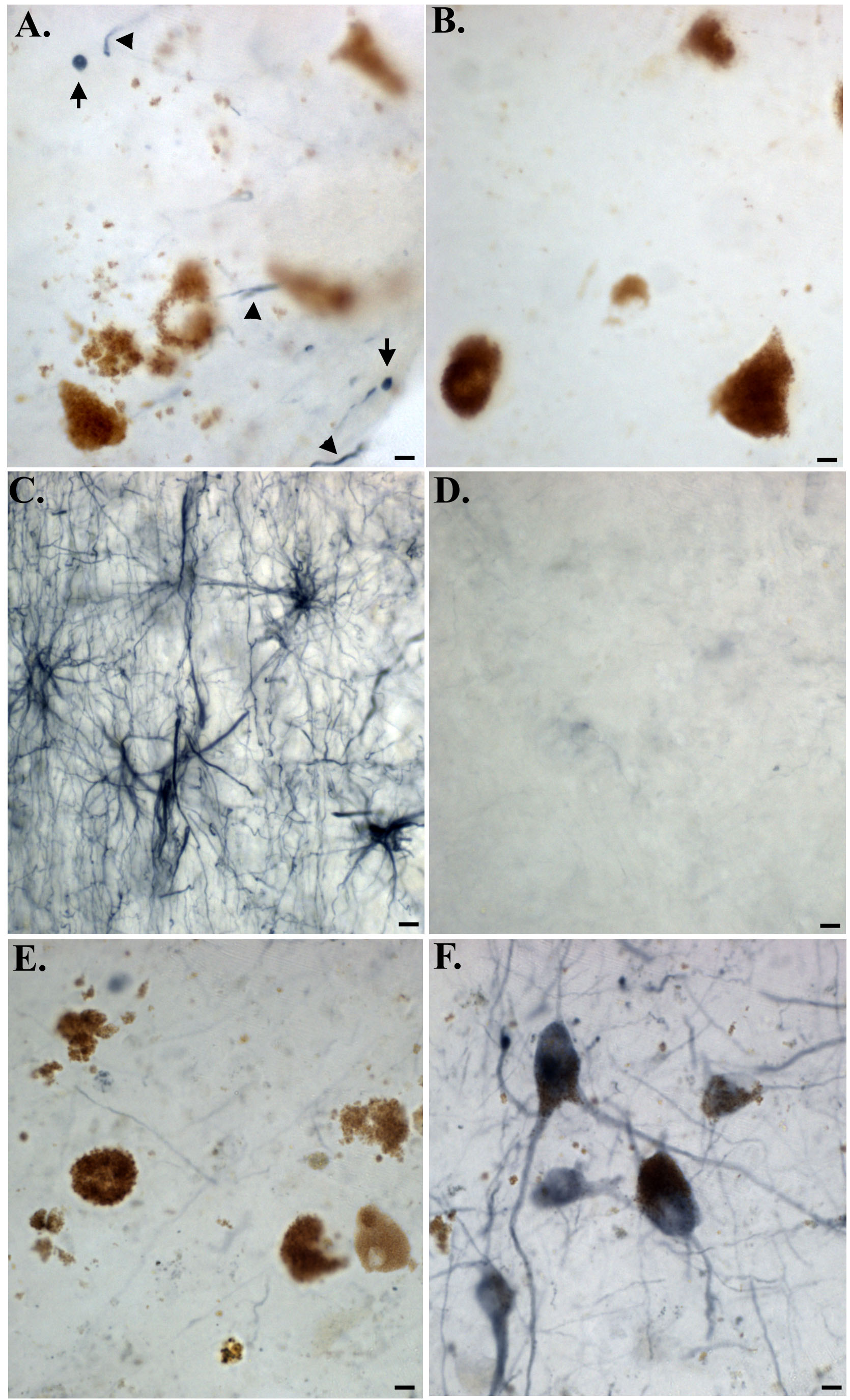


**Figure S1. Representative pathology in PD cases.** Panels A, C, and E depict images from representative PD cases, while Panels B, D, and F are from representative control cases. **(A and B):** The presence of Lewy bodies (arrows, A) and Lewy neurites in PD (arrowheads, A) that was absent in age-matched control sections (B). **(C and D):** Neuroinflammation in PD cases was revealed following the demonstration of massive gliosis that was absent in age-matched control cases (D). **(E and F):** Loss of dopaminergic neurons in PD cases was revealed following labeling with an anti-tyrosine hydroxylase antibody (E) compared to age-matched controls (F). Note also the general loss of pigmentation in PD cases (A and E). All antibody staining is shown in blue, while brown labeling depicts the presence of neuromelanin that is typical of neurons found in the SNpc. All scale bars represent 10 µm.
