# Supplementary material for: Immunolocalization of Influenza A Virus and Markers of Inflammation in the Human Parkinson's Disease Brain
Source: PLoS One. 2011 May 31;6(5):e20495. doi: 10.1371/journal.pone.0020495 (PMC3105060; doi:10.1371/journal.pone.0020495)
Supplement: Figure S2 — Relative lack of oligodendrocyte labeling in the SNpc of PD. Representation from a PD case in blue showing the relative lack of labeling using anti-Olig1 in the SNpc (B) as compared to the widespread staining observed utilizing the BeclinCCP antibody (A). Arrows in Panel B designate the few oligodendrocytes labeled by anti-Olig1 in the SNpc. Brown structures shown in Panels A and B represent neuromelanin, typical of neurons in the SNpc. Scale bars represent 10 µm. (DOC) [file pone.0020495.s002.doc]

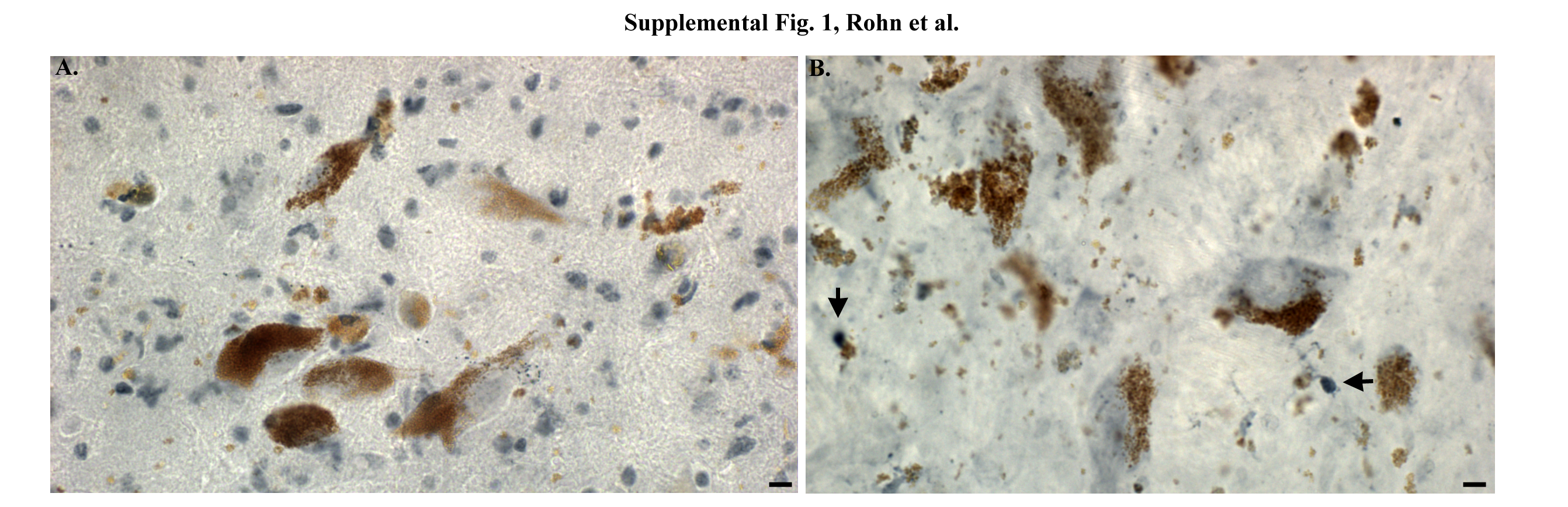


**Figure S2. Relative lack of oligodendrocyte labeling in the SNpc of PD**. Representation from a PD case in blue showing the relative lack of labeling using anti-Olig1 in the SNpc (B) as compared to the widespread staining observed utilizing the BeclinCCP antibody (A). Arrows in Panel B designate the few oligodendrocytes labeled by anti-Olig1 in the SNpc. Brown structures shown in Panels A and B represent neuromelanin, typical of neurons in the SNpc. Scale bars represent 10 µm.
