# Supplementary material for: Immunolocalization of Influenza A Virus and Markers of Inflammation in the Human Parkinson's Disease Brain
Source: PLoS One. 2011 May 31;6(5):e20495. doi: 10.1371/journal.pone.0020495 (PMC3105060; doi:10.1371/journal.pone.0020495)
Supplement: Figure S3 — Evidence for influenza A virus on macrophages within the DLB brain. Double-labeling immunofluorescence in the SNpc of a representative DLB case utilizing mAB anti-CD206, a macrophage marker (green, A) along with anti-influenza A virus labeling (35–481) (red, B) with the overlap image (yellow/orange, C). Scale bar represents 10 µm. (DOC) [file pone.0020495.s003.doc]

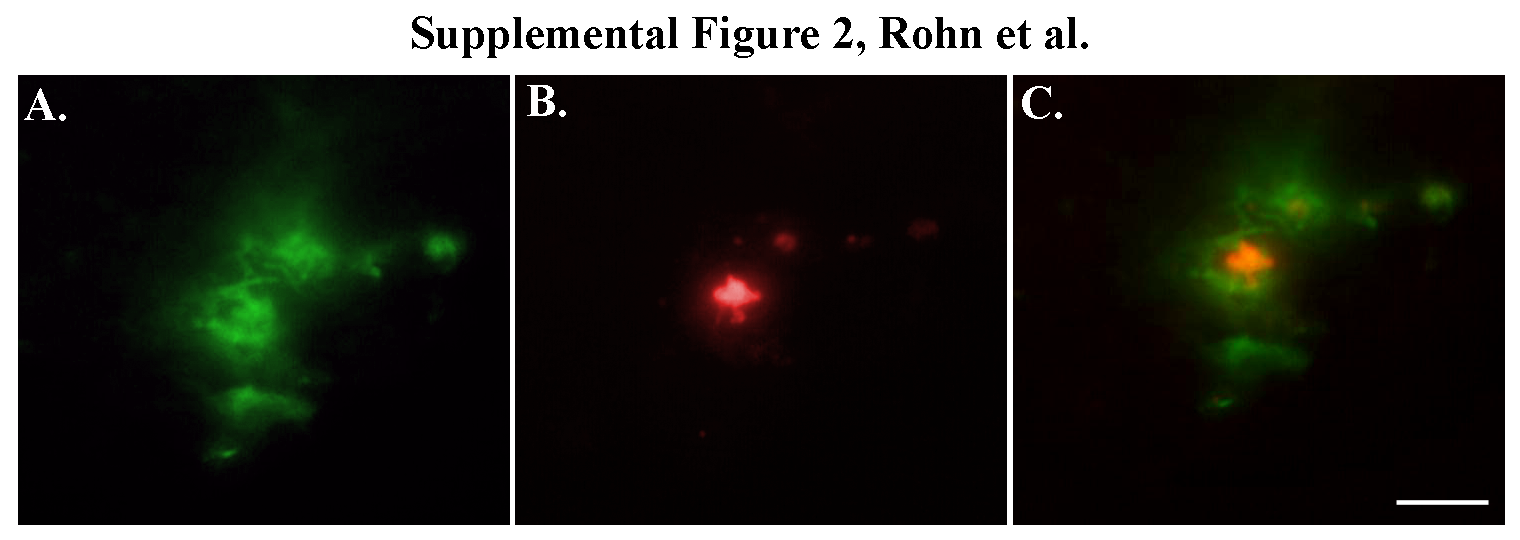


**Figure S3. Evidence for influenza A virus on macrophages within the DLB brain.** Double-labeling immunofluorescence in the SNpc of a representative DLB case utilizing mAB anti-CD206, a macrophage marker (green, A) along with anti-influenza A virus labeling (35-481) (red, B) with the overlap image (yellow/orange, C). Scale bar represents 10 µm.
