# Supplementary material for: Immunolocalization of Influenza A Virus and Markers of Inflammation in the Human Parkinson's Disease Brain
Source: PLoS One. 2011 May 31;6(5):e20495. doi: 10.1371/journal.pone.0020495 (PMC3105060; doi:10.1371/journal.pone.0020495)
Supplement: Table S1 — (DOC) [file pone.0020495.s004.doc]

**Table S1: Case Demographics for Parkinson’s Disease**

| **Case** | **Sex** | **PMI** | **NPDx1** | **Cause of Death** |
| --- | --- | --- | --- | --- |
| 1 | M | 2.25 | PD | Respiratory Failure |
| 2 | M | 3.5 | PD | Cardiopulmonary Arrest |
| 3 | F | 3 | PD | Pneumonia |
| 4 | F | 2.3 | PD | Unknown |
| 5 | M | 4.5 | PD | Cardiopulmonary Arrest |
| 6 | F | 6 | PD/AD | Respiratory Failure |
| 7 | F | 3.8 | PD/AD | Cariopulmonary Arrest |

PMI = postmortem interval; NPDx1; primary pathological diagnosis
